# Supplementary figures and images for: Epidemiological analysis reveals coral species affected by stony coral tissue loss disease present a similar epizootic progression despite differences in susceptibility and population impact
Source: PLoS One. 2026 Jan 2;21(1):e0339054. doi: 10.1371/journal.pone.0339054 (PMC12758708; doi:10.1371/journal.pone.0339054)

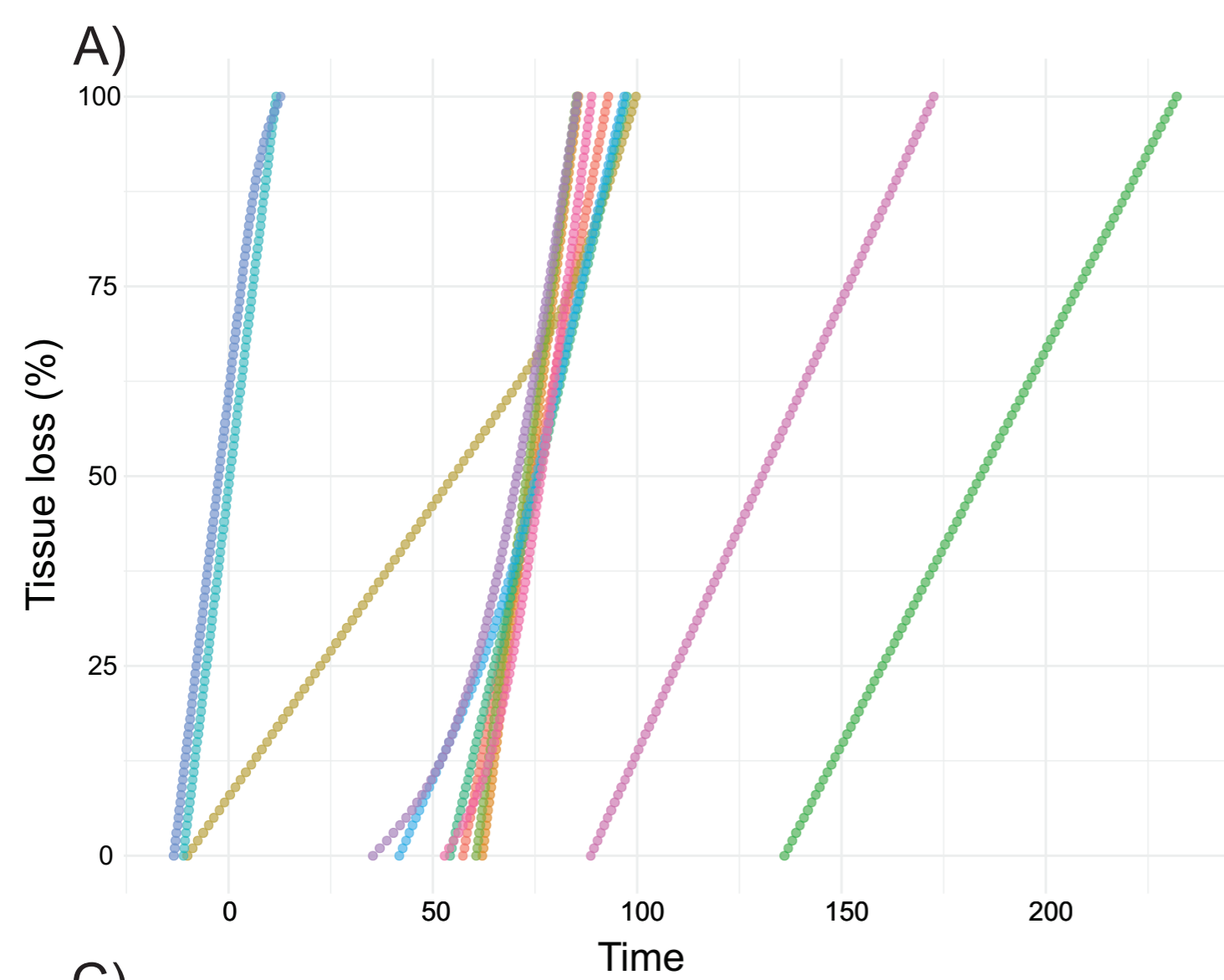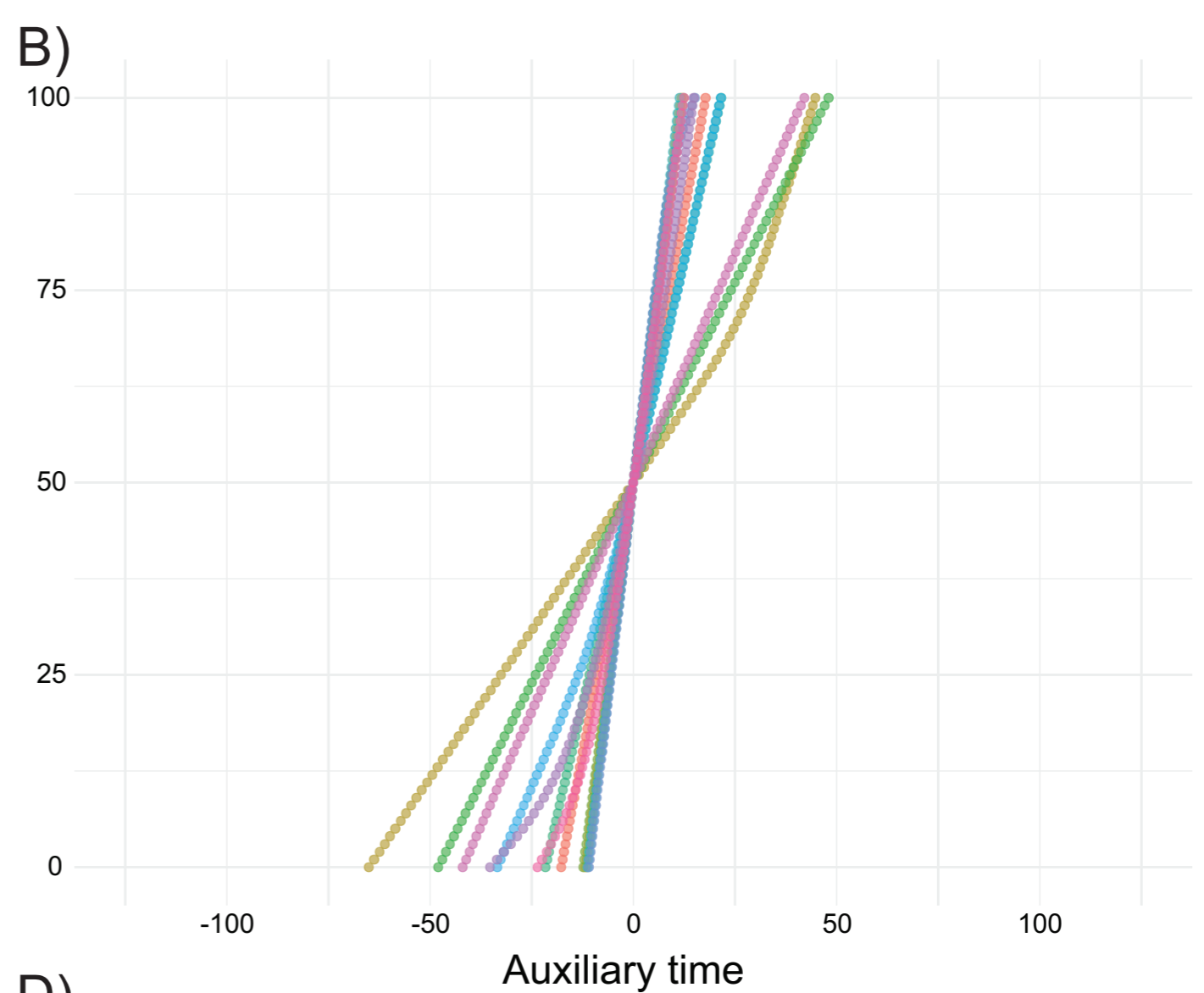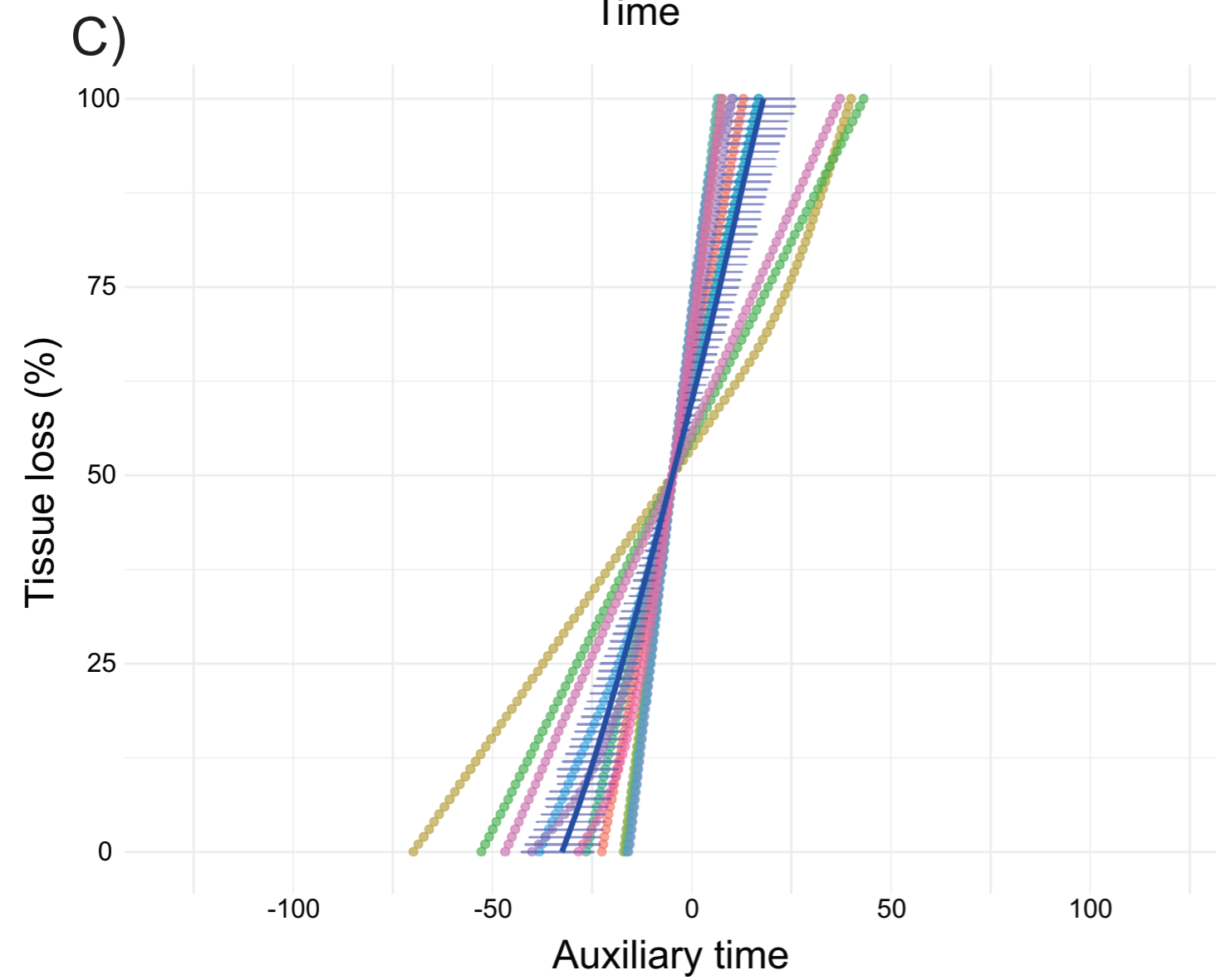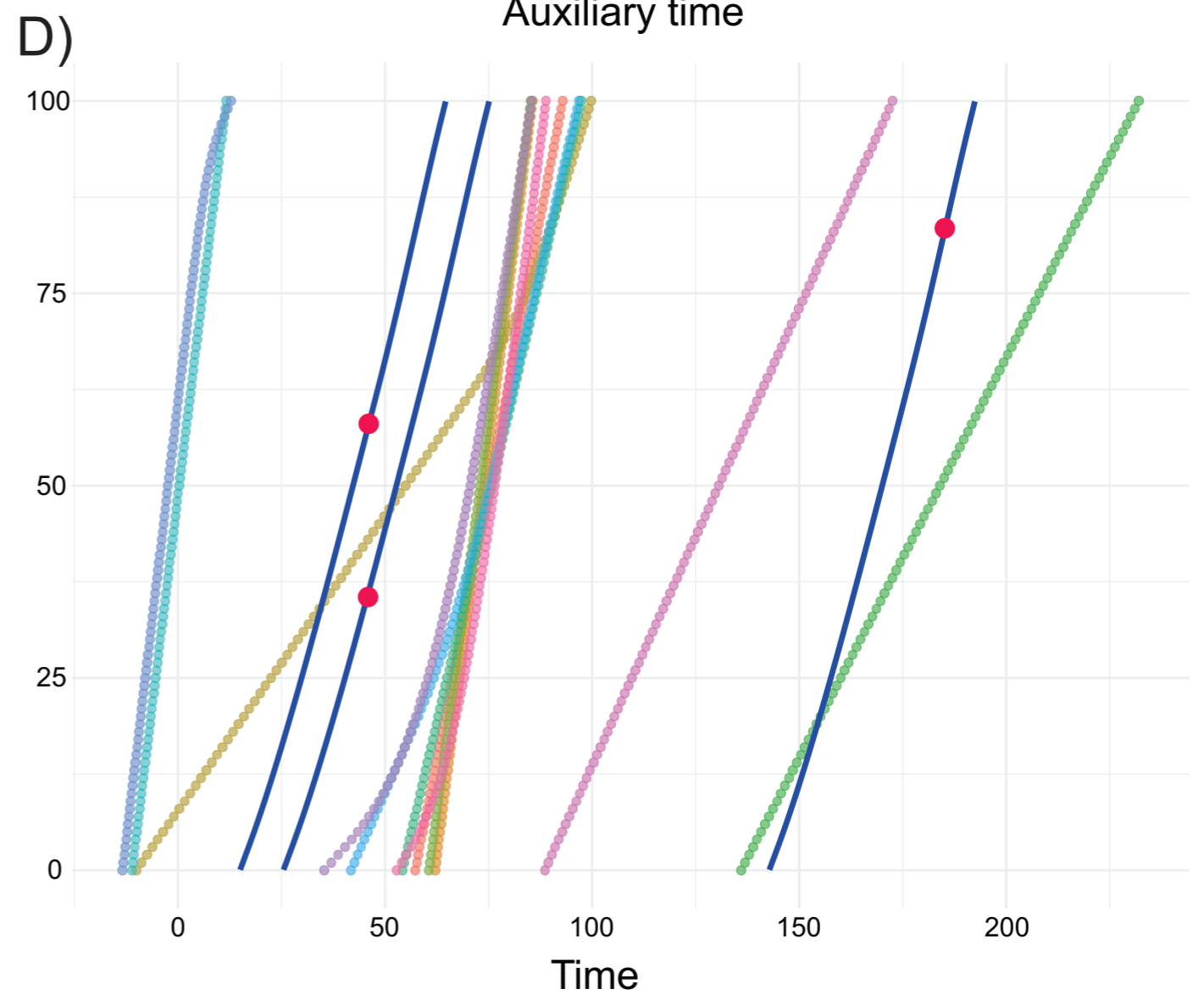

Supplement: S1 Fig — Example for P. strigosa (medium size, acute lesion progression, n = 12): A) Daily tissue loss trajectories from colonies with multiple observations were grouped by species, size category, and lesion progression type. Each colored line is an individual trajectory. B) Time was normalized around the 50% tissue loss point to align trajectories and estimate average trajectory. C) The average trajectory (blue line) and its 95% confidence interval (blue ribbon) were computed via bootstrap percentile method. D) Red dots show single-record colonies; their lesion progression was extrapolated using the reference trajectory, assuming similar dynamics. (PDF) [file pone.0339054.s001.pdf]

Day 100

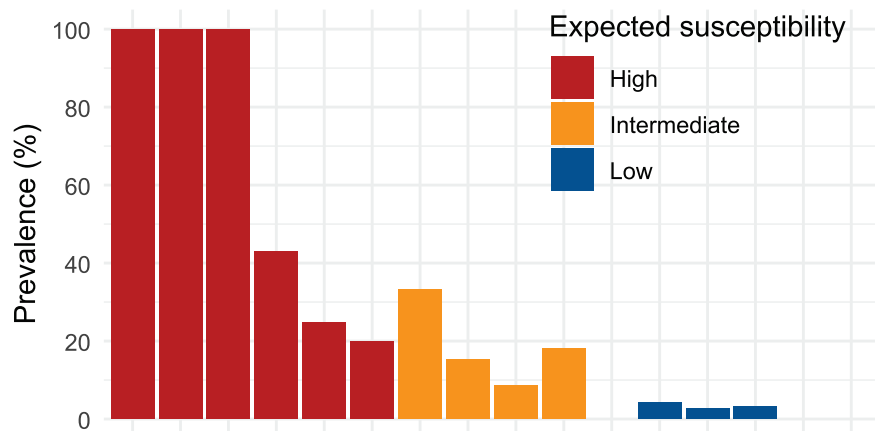

Day 200

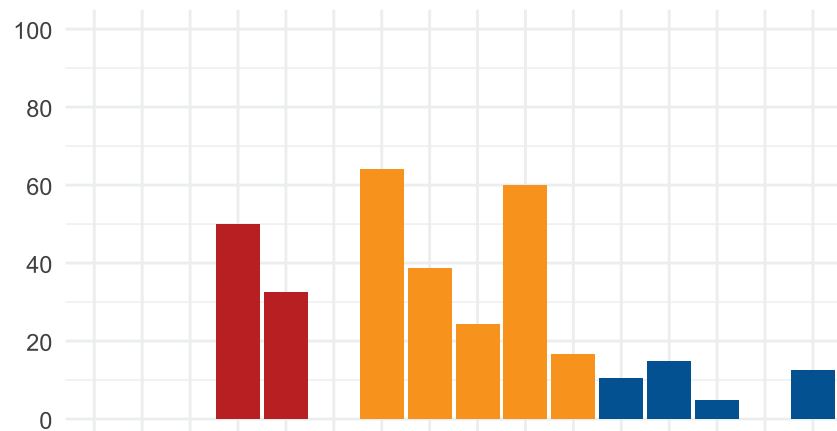

Day 300

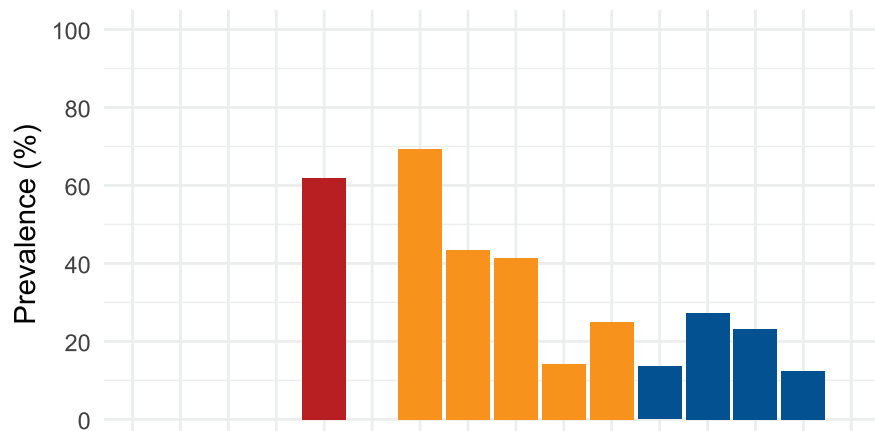

Day 400

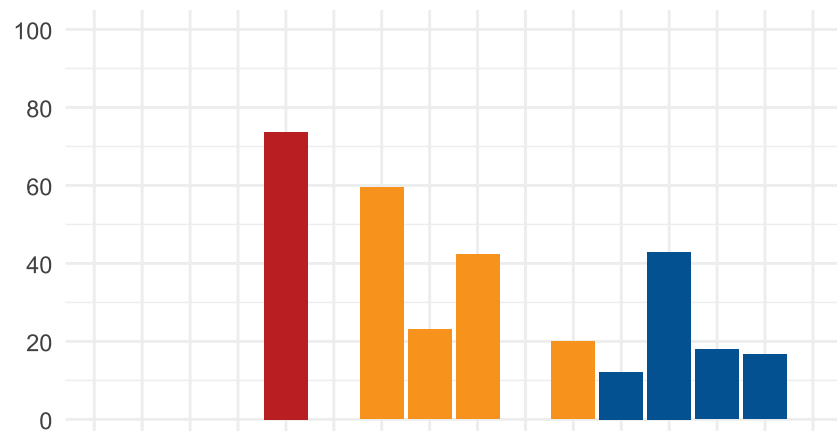

Day 500

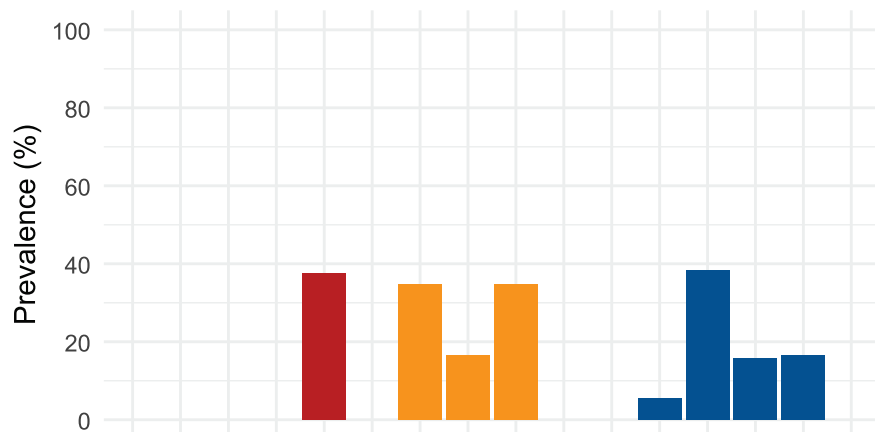

Day 600

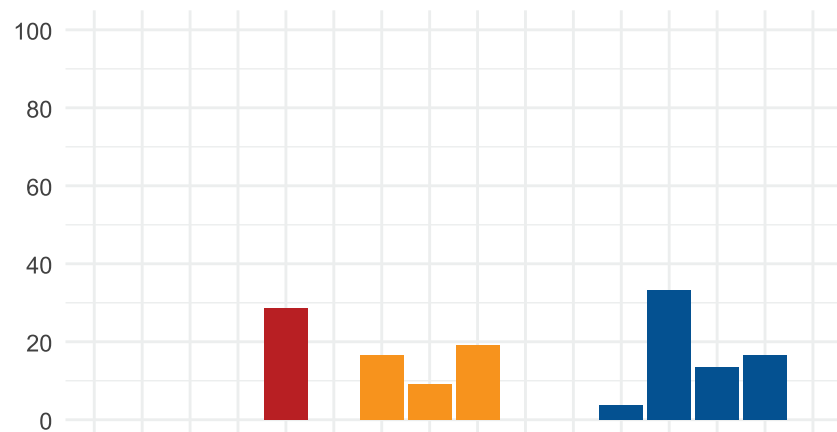

Supplement: S5 Fig — Colors show the expected susceptibility group: High (red), Intermediate (yellow), and Low (blue). Panels show that prevalence comparisons between species differed at each time point. Furthermore, the prevalence levels of species mostly failed to match the expected susceptibility patterns across the sub-sampled periods. (PDF) [file pone.0339054.s005.pdf]
