# Supplementary material for: Epidemiological analysis reveals coral species affected by stony coral tissue loss disease present a similar epizootic progression despite differences in susceptibility and population impact
Source: PLoS One. 2026 Jan 2;21(1):e0339054. doi: 10.1371/journal.pone.0339054 (PMC12758708; doi:10.1371/journal.pone.0339054)
Supplement: S3 Fig — Colored bands represent the 95% Confidence Interval. Colors indicate the expected susceptibility groups: red for high susceptibility, yellow for medium susceptibility, and blue for low susceptibility. A notable decrease in risk is observed as one moves to lower levels of expected susceptibility. However, some species, such as P. clivosa, S. siderea, and S. intersepta, display values that deviate from their expected susceptibility groups. Overall, the medium susceptibility group exhibited greater heterogeneity. (PDF) [file pone.0339054.s003.pdf]

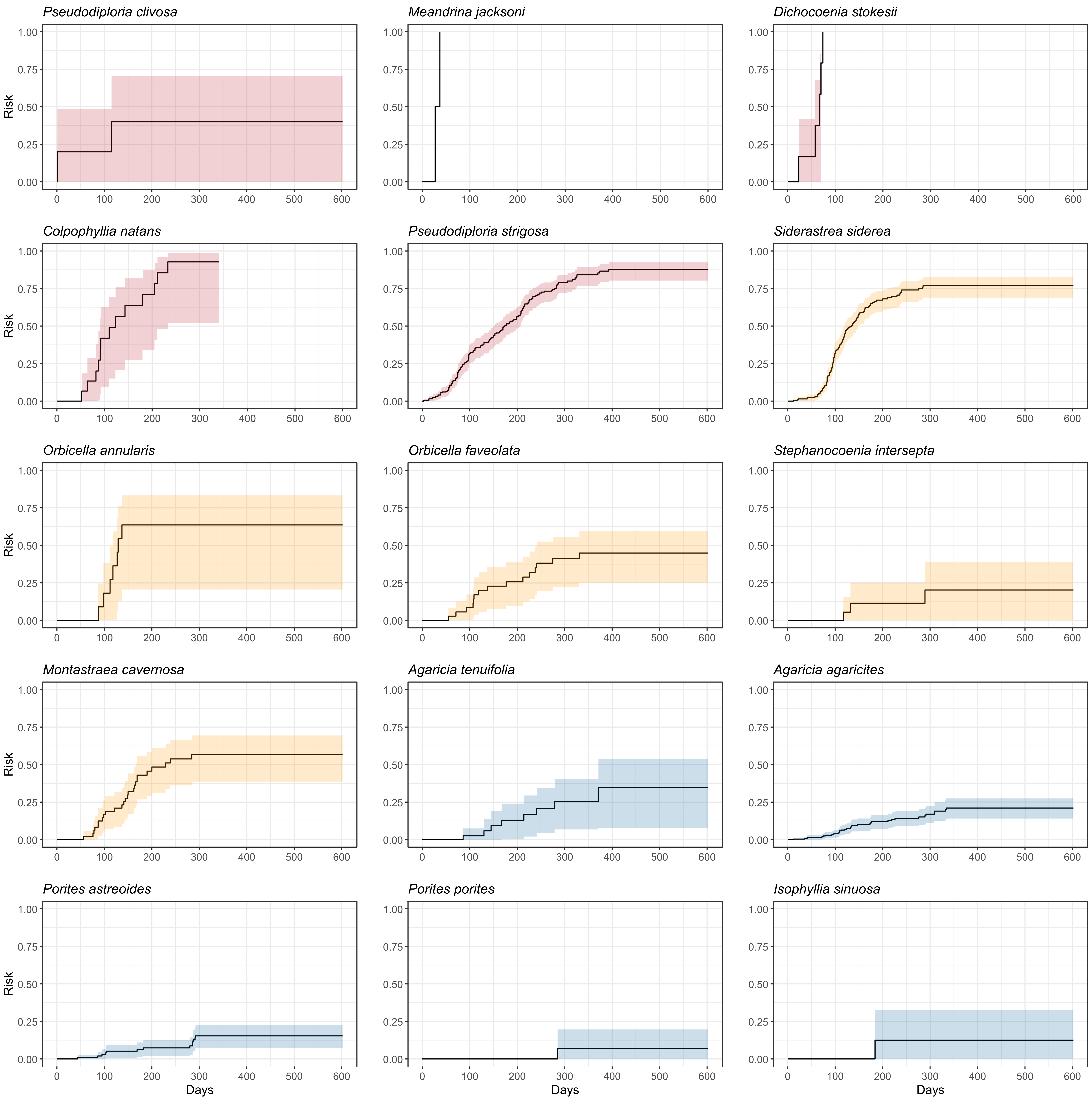

Pseudodiploria clivosa
Meandrina jacksoni
Dichocoenia stokesii
Colpophyllia natans
Pseudodiploria strigosa
Siderastrea siderea
Orbicella annularis
Orbicella faveolata
Stephanocoenia intersepta
Montastraea cavernosa
Agaricia tenuifolia
Agaricia agaricites
Porites astreoides
Porites porites
Isophyllia sinuosa
Risk
Days
0.00
0.25
0.50
0.75
1.00
0
100
200
300
400
500
600
