# Supplementary material for: Epidemiological analysis reveals coral species affected by stony coral tissue loss disease present a similar epizootic progression despite differences in susceptibility and population impact
Source: PLoS One. 2026 Jan 2;21(1):e0339054. doi: 10.1371/journal.pone.0339054 (PMC12758708; doi:10.1371/journal.pone.0339054)
Supplement: S4 Fig — Bands represent the 95% Confidence Interval. For clarity, graphs display data up to day 600 from the outbreak onset. Decreases in survival probabilities occurred beyond day 600 are depicted as vertically dashed lines. These lines do not indicate sudden drops at that point but summarize posterior mortality up to 2.8 years. (PDF) [file pone.0339054.s004.pdf]

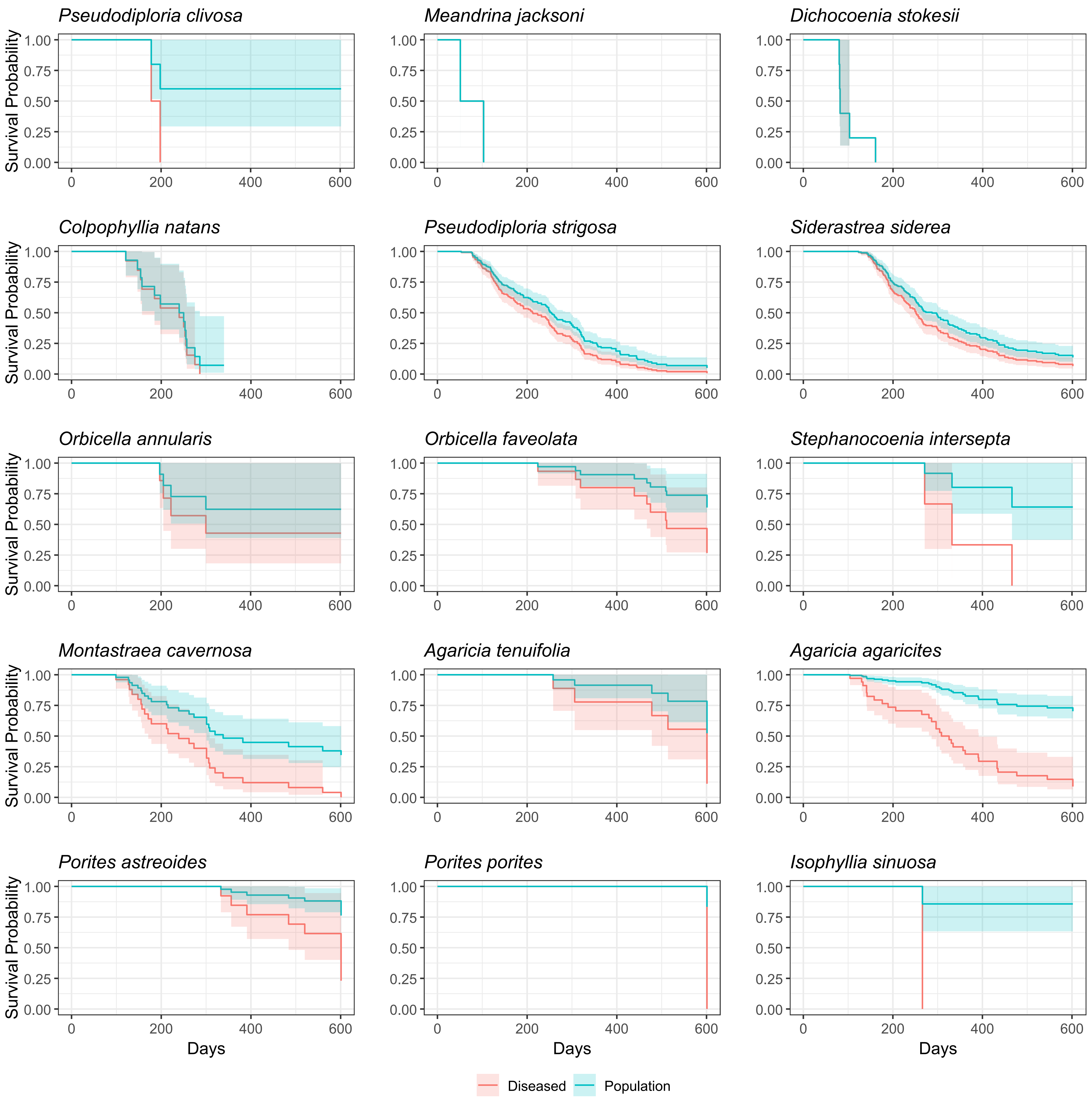
Pseudodiploria clivosa
Meandrina jacksoni
Dichocoenia stokesii
Colpophyllia natans
Pseudodiploria strigosa
Siderastrea siderea
Orbicella annularis
Orbicella faveolata
Stephanocoenia intersepta
Montastraea cavernosa
Agaricia tenuifolia
Agaricia agaricites
Porites astreoides
Porites porites
Isophyllia sinuosa
Survival Probability
Days
Diseased
Population
