# Supplementary material for: Epidemiological analysis reveals coral species affected by stony coral tissue loss disease present a similar epizootic progression despite differences in susceptibility and population impact
Source: PLoS One. 2026 Jan 2;21(1):e0339054. doi: 10.1371/journal.pone.0339054 (PMC12758708; doi:10.1371/journal.pone.0339054)
Supplement: S1 Table — (PDF) [file pone.0339054.s006.pdf]

Table S1. Frequency of use of parameters to classify species susceptibility to SCTLD.

Prevalence	Tissue Loss Rate	Mortality Rate	Survival/ Risk analysis	Transmissibility tests	Disease onset	Reference
1		1				Precht et al., 2016
1	1			1	1	FDEP, 2018
1						Walton et al., 2018
				1		Aeby et al., 2019
1						Alvarez-Filip et al., 2019
	1					Meiling et al., 2020
1	1	1				Sharp et al., 2020
1						Muller et al., 2020
1	1		1	1		Williams et al., 2020
1						Costa et al., 2021
1	1				1	Williams et al., 2021a
	1					Eaton et al., 2021
1		1	1			Spadafore et al., 2021
1						Williams et al., 2021b
1						Dahlgren et al., 2021
1						Brandt et al., 2021
1	1		1			Meiling et al., 2021
1		1				Estrada-Saldívar et al., 2021
1		1				Kolodziej et al., 2021
1						Cróquer et al., 2021
1						(Hing et al., 2022
1	1					Hayes et al., 2022
1		1				Alvarez-Filip et al., 2022
1						Johnston et al., 2023
1						Lucas et al., 2024
	1					Papke et al., 2023
1	1					Reyes-de-la-Puente et al., 2025
**23**	**10**	**6**	**3**	**3**	**2**	

References

Aeby, G. S., Ushijima, B., Campbell, J. E., Jones, S., Williams, G. J., Meyer, J. L., Häse, C., & Paul, V. J. (2019). Pathogenesis of a Tissue Loss Disease Affecting Multiple Species of Corals Along the Florida Reef Tract. Frontiers in Marine Science, 6. https://doi.org/10.3389/fmars.2019.00678

Alvarez-Filip, L., Estrada-Saldívar, N., Pérez-Cervantes, E., Molina-Hernández, A., & González-Barrios, F. J. (2019). A rapid spread of the stony coral tissue loss disease outbreak in the Mexican Caribbean. PeerJ, 2019(11). https://doi.org/10.7717/peerj.8069

Alvarez-Filip, L., González-Barrios, F. J., Pérez-Cervantes, E., Molina-Hernández, A., & Estrada-Saldívar, N. (2022). Stony coral tissue loss disease decimated Caribbean coral populations and reshaped reef functionality. Communications Biology, 5(1). https://doi.org/10.1038/s42003-022-03398-6

Brandt, M. E., Ennis, R. S., Meiling, S. S., Townsend, J., Cobleigh, K., Glahn, A., Quetel, J., Brandtneris, V., Henderson, L. M., & Smith, T. B. (2021). The Emergence and Initial Impact of Stony Coral Tissue Loss Disease (SCTLD) in the United States Virgin Islands. Frontiers in Marine Science, 8. https://doi.org/10.3389/fmars.2021.715329

Costa, S. V., Hibberts, S. J., Olive, D. A., Budd, K. A., Long, A. E., Meiling, S. S., Miller, M. B., Vaughn, K. M., Carrión, C. I., Cohen, M. B., Savage, A. E., Souza, M. F., Buckley, L., Grimes, K. W., Platenberg, R., Smith, T. B., Blondeau, J., & Brandt, M. E. (2021). Diversity and Disease: The Effects of Coral Diversity on Prevalence and Impacts of Stony Coral Tissue Loss Disease in Saint Thomas, U.S. Virgin Islands. Frontiers in Marine Science, 8. https://doi.org/10.3389/fmars.2021.682688

Cróquer, A., Weil, E., & Rogers, C. S. (2021). Similarities and Differences Between Two Deadly Caribbean Coral Diseases: White Plague and Stony Coral Tissue Loss Disease. In Frontiers in Marine Science (Vol. 8). Frontiers Media S.A. https://doi.org/10.3389/fmars.2021.709544

Dahlgren, C., Pizarro, V., Sherman, K., Greene, W., & Oliver, J. (2021). Spatial and Temporal Patterns of Stony Coral Tissue Loss Disease Outbreaks in The Bahamas. Frontiers in Marine Science, 8. https://doi.org/10.3389/fmars.2021.682114

Eaton, K. R., Landsberg, J. H., Kiryu, Y., Peters, E. C., & Muller, E. M. (2021). Measuring Stony Coral Tissue Loss Disease Induction and Lesion Progression Within Two Intermediately Susceptible Species, Montastraea cavernosa and Orbicella faveolata. Frontiers in Marine Science, 8. https://doi.org/10.3389/fmars.2021.717265

Estrada-Saldívar, N., Quiroga-García, B. A., Pérez-Cervantes, E., Rivera-Garibay, O. O., & Alvarez-Filip, L. (2021). Effects of the Stony Coral Tissue Loss Disease Outbreak on Coral Communities and the Benthic Composition of Cozumel Reefs. Frontiers in Marine Science, 8. https://doi.org/10.3389/fmars.2021.632777

Florida Department of Environmental Protection (FDEP). Stony Coral Tissue Loss Disease (SCTLD) Case Definition. 2018. Available from: https://floridadep.gov/sites/default/files/Copy%20of%20StonyCoralTissueLossDisease_CaseDefinition%20final%2010022018.pdf

Hayes, N. K., Walton, C. J., & Gilliam, D. S. (2022). Tissue loss disease outbreak significantly alters the Southeast Florida stony coral assemblage. Frontiers in Marine Science, 9. https://doi.org/10.3389/fmars.2022.975894

Johnston, M. A., Studivan, M. S., Enochs, I. C., Correa, A. M. S., Besemer, N., Eckert, R. J., Edwards, K., Hannum, R., Hu, X., Nuttall, M., O'Connell, K., Palacio-Castro, A. M., Schmahl, G. P., Sturm, A. B., Ushijima, B., & Voss, J. D. (2023). Coral disease

outbreak at the remote Flower Garden Banks, Gulf of Mexico. Frontiers in Marine Science, 10. https://doi.org/10.3389/fmars.2023.1111749

Kolodziej, G., Studivan, M. S., Gleason, A. C. R., Langdon, C., Enochs, I. C., & Manzello, D. P. (2021). Impacts of Stony Coral Tissue Loss Disease (SCTLD) on Coral Community Structure at an Inshore Patch Reef of the Upper Florida Keys Using Photomosaics. Frontiers in Marine Science, 8. https://doi.org/10.3389/fmars.2021.682163

Lee Hing, C., Guifarro, Z., Dueñas, D., Ochoa, G., Nunez, A., Forman, K., Craig, N., & McField, M. (2022). Management responses in Belize and Honduras, as stony coral tissue loss disease expands its prevalence in the Mesoamerican reef. Frontiers in Marine Science, 9. https://doi.org/10.3389/fmars.2022.883062

Lucas, M. Q., Collazo Roman, D. L., Mercado, M. A., Fain, E. J., Toledo-Rodríguez, D. A., & Weil, E. (2024). Stony coral tissue loss disease (SCTLD) induced mass mortality at Arecibo, Puerto Rico. Marine Biodiversity, 54(1). https://doi.org/10.1007/s12526-023-01393-6

Meiling, S., Muller, E. M., Smith, T. B., & Brandt, M. E. (2020). 3D Photogrammetry Reveals Dynamics of Stony Coral Tissue Loss Disease (SCTLD) Lesion Progression Across a Thermal Stress Event. Frontiers in Marine Science, 7. https://doi.org/10.3389/fmars.2020.597643

Meiling, S. S., Muller, E. M., Lasseigne, D., Rossin, A., Veglia, A. J., MacKnight, N., Dimos, B., Huntley, N., Correa, A. M. S., Smith, T. B., Holstein, D. M., Mydlarz, L. D., Apprill, A., & Brandt, M. E. (2021). Variable Species Responses to Experimental Stony Coral Tissue Loss Disease (SCTLD) Exposure. Frontiers in Marine Science, 8. https://doi.org/10.3389/fmars.2021.670829

Muller, E. M., Sartor, C., Alcaraz, N. I., & van Woesik, R. (2020). Spatial Epidemiology of the Stony-Coral-Tissue-Loss Disease in Florida. Frontiers in Marine Science, 7. https://doi.org/10.3389/fmars.2020.00163

Papke, E., Carreiro, A., Dennison, C., Deutsch, J. M., Isma, L. M., Meiling, S. S., Rossin, A. M., Baker, A. C., Brandt, M. E., Garg, N., Holstein, D. M., Traylor-Knowles, N., Voss, J. D., & Ushijima, B. (2023). Stony coral tissue loss disease: a review of emergence, impacts, etiology, diagnostics, and intervention. In Frontiers in Marine Science (Vol. 10). Frontiers Media SA. https://doi.org/10.3389/fmars.2023.1321271

Precht, W. F., Gintert, B. E., Robbart, M. L., Fura, R., & Van Woesik, R. (2016). Unprecedented Disease-Related Coral Mortality in Southeastern Florida. Scientific Reports, 6. https://doi.org/10.1038/srep31374

Reyes-de-la-Puente, G., Jordán-Garza, A. G., Morales-Ramírez, B. A., Rodríguez-Villalobos, J. C., & Rodríguez-Martínez, R. E. (2025). A 2018 epizootic of a tissue-loss disease in the Southwestern Gulf of Mexico: implications for coral health and

conservation. Coral Reefs, 44(2), 689–700. https://doi.org/10.1007/s00338-025-02636-7

Sharp, W. C., Shea, C. P., Maxwell, K. E., Muller, E. M., & Hunt, J. H. (2020). Evaluating the small-scale epidemiology of the stony-coral -tissue-loss-disease in the middle Florida Keys. PLoS ONE, 15(11 November). https://doi.org/10.1371/journal.pone.0241871

Spadafore, R., Fura, R., Precht, W. F., & Vollmer, S. V. (2021). Multi-Variate Analyses of Coral Mortality From the 2014–2015 Stony Coral Tissue Loss Disease Outbreak Off Miami-Dade County, Florida. Frontiers in Marine Science, 8. https://doi.org/10.3389/fmars.2021.723998

Walton, C. J., Hayes, N. K., & Gilliam, D. S. (2018). Impacts of a regional, multi-year, multi-species coral disease outbreak in Southeast Florida. Frontiers in Marine Science, 5(SEP). https://doi.org/10.3389/fmars.2018.00323

Williams, L., Smith, T. B., Burge, C. A., & Brandt, M. E. (2020). Species-specific susceptibility to white plague disease in three common Caribbean corals. Coral Reefs, 39(1), 27–31. https://doi.org/10.1007/s00338-019-01867-9

Williams, S. D., Walter, C. S., & Muller, E. M. (2021a). Fine Scale Temporal and Spatial Dynamics of the Stony Coral Tissue Loss Disease Outbreak Within the Lower Florida Keys. Frontiers in Marine Science, 8. https://doi.org/10.3389/fmars.2021.631776

Williams, S. M., García-Sais, J., & Sabater-Clavell, J. (2021b). Prevalence of Stony Coral Tissue Loss Disease at El Seco, a Mesophotic Reef System off Vieques Island, Puerto Rico. Frontiers in Marine Science, 8. https://doi.org/10.3389/fmars.2021.668669
