# Supplementary material for: Epidemiological analysis reveals coral species affected by stony coral tissue loss disease present a similar epizootic progression despite differences in susceptibility and population impact
Source: PLoS One. 2026 Jan 2;21(1):e0339054. doi: 10.1371/journal.pone.0339054 (PMC12758708; doi:10.1371/journal.pone.0339054)
Supplement: S5 Table — Status codes: Recently dead, denotes recently denuded skeleton; Film 1 is a scarce microbial/algal film; Film 2 is a well-stablished biofilm; Film 3 incipient turf algae mats; Turf Algae, denser turf algae mats; Macro Algae, macroalgal patches identifiable to genus level. (n) is the number of colonies analyzed to time calculations. CI = Confidence Interval of the 95%. (PDF) [file pone.0339054.s010.pdf]

Table S5. Mean time of change from apparently healthy tissue adjacent to any lesion to each stage of colonization. CI: Confidence Interval of the 95%.

**Initial condition**	**Status**	**n**	**Time (Days)**	**CI (± days)**
Healthy	Recently dead	8	4.3	1.0
Healthy	Film 1	12	7.4	1.5
Healthy	Film 2	54	14.7	0.9
Healthy	Film 3	8	40.1	18.6
Healthy	Turf Algae	74	54.9	5.1
Healthy	Macro Algae	1	99.0	-
**Total**		**157**		
