# Supplementary material for: Epidemiological analysis reveals coral species affected by stony coral tissue loss disease present a similar epizootic progression despite differences in susceptibility and population impact
Source: PLoS One. 2026 Jan 2;21(1):e0339054. doi: 10.1371/journal.pone.0339054 (PMC12758708; doi:10.1371/journal.pone.0339054)
Supplement: S2 Fig — Process illustrated with two colonies of P. strigosa. Dual view per colony: natural appearance (top) and colonization stage overlays (bottom). Legend at the lower right corner. Colonization stages are arranged outward from the living tissue and delineated by distinct line patterns in the following order: Recently dead (almost pure white coloration), Film 1 (incipient microbial film), Film 2 (well-established microbial biofilm; denser algal films with more saturated hues), Film 3 (incipient algal mat; filamentous algae beginning to establish, with possible sediment entrapment), Turf algae (denser mats of juvenile macroalgae or filamentous algae), and Macroalgae (advanced macroalgal growth, often identifiable to genus level). (PDF) [file pone.0339054.s002.pdf]

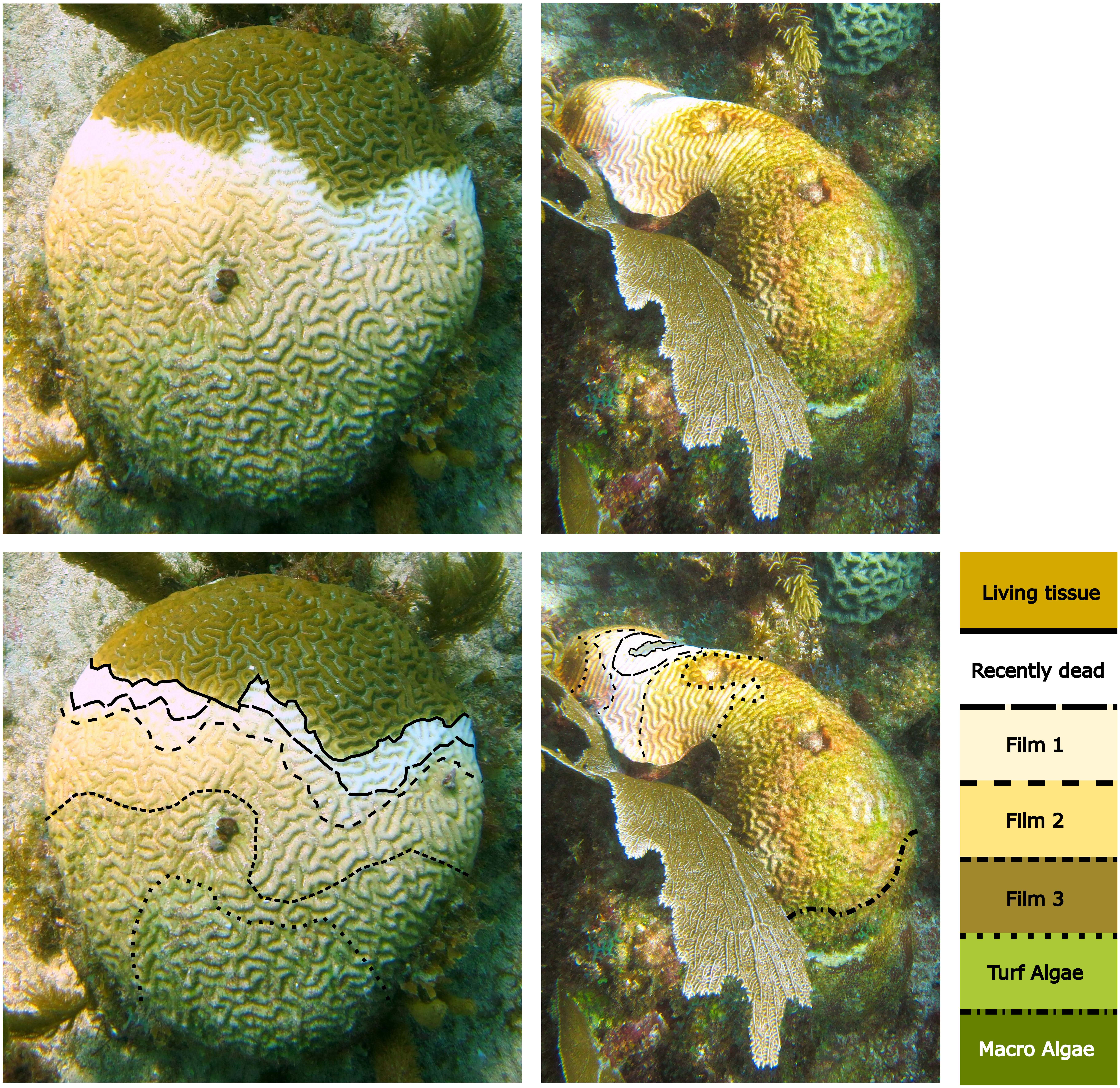
Living tissue
Recently dead
Film 1
Film 2
Film 3
Turf Algae
Macro Algae
