# Supplementary material for: Epidemiological analysis reveals coral species affected by stony coral tissue loss disease present a similar epizootic progression despite differences in susceptibility and population impact
Source: PLoS One. 2026 Jan 2;21(1):e0339054. doi: 10.1371/journal.pone.0339054 (PMC12758708; doi:10.1371/journal.pone.0339054)
Supplement: Attachment — Submitted filename: PLOSOne SCTLD epi.docx [file pone.0339054.s012.docx]

This paper investigates the susceptibility of 16 species of stony corals based on epizootic and Kaplan-Meier curves, giving a picture of the incidence and spread over time that is much more accurate and comprehensive than punctual prevalence.

One concern I have is regarding how many colonies tissue loss was calculated based on a single observation and/or on extrapolating from algal colonization. Did this occur pretty evenly between species/ susceptibility classes, or did it disproportionally affect one or more and might have impacted those comparisons?

I am interested to see the peaks in transmission following mortality, and speculation about the role of non-primary pathogens and dysbiosis. Could this explain the variety of histological lesions found in outbreaks after the early Florida investigation? Work et al. “Pathology of lesions in corals from the US Virgin Islands after emergence of stony coral tissue loss disease” describes a variety of lesions that largely do not match the ones found in the initial Landsburg paper.

Line 107: write 17^th^ century instead of using Roman numerals.

Lines 110-112: reads awkwardly, suggest: “They help to determine the progression, development and magnitude of an epidemic or epizootic, distinguish epizootic from enzootic phases, and can be compared with potentially correlated factors.”

Line 150: How many total surveys?

Write out “approximately” instead of using a tilde.

Line 150-153: “Both sites are 5 m deep, and the sites are separated by 1,100m” Or put the distance the sites are apart at the beginning of the paragraph “at two sites afflicted by SCTLD and 1,100 m apart in the Puerto Morales reef..”

Line 160: change to “During each subsequent survey”

Line 161: How did you determine that SCTLD was the cause of tissue loss in any given colony? Was all observed tissue loss assumed to be due to SCTLD? Did you observe any loss to predation or other causes?

Line 165: “focused”; maintain verb tense.

Line 197: change “and” to “that” did not…

Line 201/S5: How many species are included in the extrapolation of time since tissue loss using algal growth, and could this vary with different types/rugosity of skeleton between species?

Fig. 2, 3: The labels and numbers on these are blurry and very difficult to read in the PDF, although the downloaded figure is sharp. If this will be physically printed or published as a PDF a higher resolution image is needed.

Line 608: change to “This is congruent with the finding that epizootics of this syndrome may not be dependent on colony density”.

Line 614: precisely? Or do you mean primarily.

Line 629: Don’t start the sentence with “and”; either “Additionally, histo…” or Just “Histopathologic analysis…”

Line 652:”is not against” here is vague; do you mean “is not incompatible with” and “large” rather than “big”.
