# Supplementary material for: Epidemiological analysis reveals coral species affected by stony coral tissue loss disease present a similar epizootic progression despite differences in susceptibility and population impact
Source: PLoS One. 2026 Jan 2;21(1):e0339054. doi: 10.1371/journal.pone.0339054 (PMC12758708; doi:10.1371/journal.pone.0339054)
Supplement: Attachment — Submitted filename: Response to Reviewers.docx [file pone.0339054.s014.docx]

**Response to Academic Editor:**

**Academic Editor recommendations:**

**1. Please ensure that your manuscript meets PLOS ONE's style requirements, including those for file naming.**

We have reviewed the PLOS ONE style requirements and ensured that they are followed in the manuscript, including file naming.

**2. Please provide an amended statement that declares *all* the funding or sources of support (whether external or internal to your organization) received during this study, as detailed online in our guide for authors at http://journals.plos.org/plosone/s/submit-now. Please also include the statement “There was no additional external funding received for this study.” in your updated Funding Statement. Please include your amended Funding Statement within your cover letter. We will change the online submission form on your behalf.**

I have included the founding statement at the end of the cover letter, including the requested information.

**3. We note that there is identifying data in the Supporting Information file <S2 Table>. Due to the inclusion of these potentially identifying data, we have removed this file from your file inventory. Prior to sharing human research participant data, authors should consult with an ethics committee to ensure data are shared in accordance with participant consent and all applicable local laws.**

I appreciate the comment; however, it appears to be a misunderstanding. Table S2 contains a bibliographic summary of scientific publications related to coral disease research. It includes only the information necessary for citation purposes, such as authors' names, publication title, year, journal name, and similar details. This work does not involve human subjects.

**4. If the reviewer comments include a recommendation to cite specific previously published works, please review and evaluate these publications to determine whether they are relevant and should be cited. There is no requirement to cite these works unless the editor has indicated otherwise.**

Thank you very much. We have considered including additional citations where appropriate.

**Response to Reviewers:**

**Reviewer #1**

**Overview: Authors attempt to describe temporal aspects of SCTLD in Mexican reefs. I like what authors are trying to do, but their approach seems overly convoluted and difficult to understand. Unless we have more details on how they did their surveys, estimates of underlying populations at risk, it becomes difficult to sort out potential confounding factors (like survey effort or number of animals available to observe) that could sway the results. The presentation of material is also garbled and does not make sense at least to this reviewer. There is definitely something interesting here but authors need to clarify, condense, and simplify to get their point across. I sense that the main points of the paper could be captured with Figures 2 and 4 with everything else deleted to simplify. Bottom line, you could get a good idea of interspecies susceptibility by plotting epidemic curves and calculating case fatality rate (the higher, the more susceptible the species). This would be cleaner and simpler to present to get your point across without a lot of convolution.**

We sincerely thank the reviewer for their thoughtful and constructive comments. In response, we have carefully revised and substantially edited the Methods section to clarify the survey design, sampling effort, and estimation of populations at risk. We also reviewed the entire manuscript to improve clarity and flow, aiming to make our approach more accessible. Please refer to your and the other reviewer's specific comments for details on how we have amended the manuscript.

We appreciate the suggestion to focus on Figures 2 and 4, which indeed highlight key aspects of susceptibility at population and community levels. However, we consider the additional analyses essential to support a more robust and biologically grounded interpretation. In coral disease studies, susceptibility is often inferred from prevalence alone, which overlooks the temporal progression of outbreaks, survival dynamics, and epidemic risk. Our approach, based on epidemic curves and Kaplan-Meier survival analysis, was designed to address these limitations and evaluate susceptibility in a temporally explicit framework.

Although this may increase the apparent complexity of the work, we consider it necessary to reflect the biological and epidemiological realities of SCTLD. While we acknowledge the communicative value of case fatality rate, we believe our framework already offers a more comprehensive representation of disease severity and susceptibility without calculating CFR separately.

**Line 79: Punctual prevalence is redundant. Just state prevalence which is percent of diseased animals at a point in time. Incidence is change in prevalence over time.**

Thank you. We agree and have changed to ‘prevalence’ as suggested.

**Lines 92-97: Difficult here to understand what you are saying. I suspect something along the following?: "Illustrative of this concept is the study by Dahlgren et al. [24] showing changes in prevalence of SCTLD by species over time."**

Thank you. We have reworded this section (lines 90 to 96) to enhance clarity and better convey the intended argument.

**Lines 104-105. Delete. It is obvious that a single sampling will not capture changes over time.**

Deleted as suggested.

**LInes 158-163: More detail on survey methodology would help here to assess validity of finding. A key aspect of temporal disease studies is implementing consistent effort spatially. Were permanent transects used and surveyed over time? Did you mark colonies? As written, it is difficult to say whether, for example, increased prevalence for a particular species was due to surveys with more of that species at a particular time point. It seems all this could be greatly simplified to present disease incidence by species to give a reader a sense of how disease varies between hosts coupled with case fatality rate for those colonies for which you have temporal observations. The latter would also address just how accurate Table 1 is for your region.**

**Lines 164-166: Were these colonies marked and followed over time? How were individual colonies recognized over time?**

The two previous comments will be answered below.

We thank the reviewer for these valuable observations. We have expanded the methods section to explain these key aspects of data collection. This more detailed description is between lines 155 – 170, as follows:

“At Bocana, four sampling points were established: B1 (453 m²), B2 (19.5 m²), B3 (140 m2), and B4 (60 m²). At Picudas, three points were defined: P1 (435 m²), P2 (12 m²) and P3 (9 m²). These polygons were delimited by reef topography and visibility constraints, but remained fixed throughout the study. Despite differences in size, consistent effort was applied within each polygon to document all visible coral colonies across species during each visit through photographic surveys.

Initially, monitoring was focused on spatial disease patterns in *Pseudodiploria strigosa*, as part of a previous study on SCTLD dynamics [26]. However, during those surveys, photographic records were systematically taken of all visible coral colonies within the same sampling areas, regardless of species. Each colony was photographed individually during every monitoring event, with multiple angles captured depending on its size and structural complexity. These images allowed us to track colonies over time and retrospectively assess their health status (healthy, diseased, or dead), visually estimate the percentage of dead tissue caused by SCTLD or previous mortality, and classify lesion progression type (acute, subacute, or chronic, sensu Work & Aeby [46]). Additionally, the maximum diameter of each colony at the onset of the study was measured using ImageJ (1.52a), based on scale markers included in the photographs.”

**Lines 190-196: I don't understand. Fig. S1 is implying each colony has 2 observations but you are talking about 1 monitoring date.**

**Fiure S1. This caption and figure makes no sense to this reviewer. I just don't understand how you can calculate rate of tissue loss over time from a single observation.**

**Lines 197-205: This reads like Greek to me.**

The three previous comments will be answered below.

Thank you for your comments. This part of the method aims to estimate the onset of lesion signs and the timing of total tissue loss in colonies with only one recorded observation. To improve clarity, we have revised the manuscript text and restructured it as follows (now in lines 209 – 238):

“For colonies with only one recorded observation of tissue loss, we acknowledge that direct estimation of lesion progression is not possible. To avoid excluding these cases, we implemented two approaches based on biologically informed assumptions and empirical reference data. These methods aim to reconstruct plausible trajectories while accounting for uncertainty, as detailed in points III and IV.

III.	The third approach was implemented for diseased colonies with only one recorded instance of tissue loss. These cases occurred either because the colonies were too small to relocate or because they died and were likely rapidly overgrown by algae, resulting in their capture as diseased during only one survey (n = 130). To address this limitation, we assumed that these individuals followed a similar trajectory to others of the same species, size category (small: <33 cm; medium: >33 to 66 cm; large: >66 to <100 cm; and extra-large: >100 cm), and lesion progression type. Based on this assumption, we first calculated individual tissue loss trajectories from colonies with sufficient temporal data, grouped by species, size category, and lesion progression type. From these trajectories, we derived an average daily tissue loss rate, which was then used to interpolate the percentage of tissue loss for colonies with only one recorded observation. This allowed us to estimate the full progression trajectory, including onset and extent of tissue loss, for single-observation cases. This procedure is illustrated in S1 Fig. A 95% confidence interval for each extrapolated trajectory was computed using the bootstrap percentile method and is provided for reference (S4 Table).

IV.	For diseased colonies with only one tissue mortality record that could not be matched to any of the above categories of species, size, and lesion progression (n = 88), we estimated their individual tissue loss trajectories based on the colonization stage of the exposed skeleton. These stages (recent mortality, algal/microbial film, turf algae, and macroalgae) follow a consistent post-mortality succession (S2 Fig). Each stage was associated with an average transition time, calculated from a reference subsample of 157 colonies (S5 Table). Using this information, we inferred when each skeletal region was covered by living tissue. Colonies displaying multiple colonization stages provided several time points along the lesion progression. Depending on the number of inferred points, we then applied either a GAMM or a linear interpolation method (as in cases I and II, respectively) to reconstruct the full trajectory of tissue loss, from lesion onset to complete mortality.”

And the S1 Figure legend accordingly (lines 944 – 951):

“**S1 Fig. Estimation of lesion progression for colonies with only one recorded tissue loss value.** Example for *P. strigosa* (medium size, acute lesion progression, n = 12): A) Daily tissue loss trajectories from colonies with multiple observations were grouped by species, size category, and lesion progression type. Each colored line is an individual trajectory. B) Time was normalized around the 50% tissue loss point to align trajectories and estimate average trajectory. C) The average trajectory (blue line) and its 95% confidence interval (blue ribbon) were computed via bootstrap percentile method. D) Red dots show single-record colonies; their lesion progression was extrapolated using the reference trajectory, assuming similar dynamics.”

**Figure S2: Seems all this could be simplified to something like acute, subacute and chronic tissue loss. I have a hard time distinguishing all the other categories you are stating**

Thank you very much for your comment. In this case, the categories correspond to colonization stages. Although there is a close relationship between these colonization stages and the rate of tissue loss, we want to relate time to the cover sequence. Therefore, we have retained the category structure explained in the legend for Figure S2 but revised both the legend and the figure itself to improve clarity and visual interpretation.

**Lines 169-209: Seems all this could be considerably simplified and condensed to the following: Calculation of tissue loss rate over 2 or 3 time points using linear models. Attempting to calculate tissue loss rate from single observations is stretching it and would suggest delete those two parts (lines 197-205).**

Thank you for your comment. We understand the concern regarding inference from single observations. However, our goal was not to calculate tissue loss rates directly from isolated data points, but rather to reconstruct plausible progression trajectories using systematic, biologically informed methods. For colonies with only one recorded observation, we relied on patterns derived from individuals of the same species, size category, and lesion type (approach III), or from the colonization stage of the exposed skeleton (approach IV), both of which are grounded in empirical data. These methods allowed us to estimate onset and extent of tissue loss in a reproducible and transparent way, while acknowledging uncertainty through confidence intervals (S4 & S5 tables). We believe this approach adds value by recovering otherwise lost information and enabling inclusion of these cases in broader epidemiological analyses. Nonetheless, we have revised the text to improve clarity and emphasize the assumptions and limitations involved. The clarified text is now found between lines 180 and 242, cited above. On the other hand, we have added a note of methodological considerations and limitations at the end of the discussion where we include this point (lines 649 – 656):

“For colonies with only one recorded observation of tissue loss, inference-based approaches (III and IV, see methods) were applied transparently, incorporating ecological indicators and uncertainty. Among these single-record cases, the distribution across susceptibility groups was approximately balanced (57% high, 44.5% intermediate, 62% low; relative to diseased colonies in each group), suggesting no systematic bias toward any group. A more influential factor was colony size: 61% of these cases corresponded to small colonies. We consider that this approach adds value by recovering information that would otherwise be excluded and by enabling the inclusion of these cases in broader epidemiological analyses”

**Line 215: Do you mean: "These curves were computed by species and by species grouped to expected susceptibility (high,intermediate and low) (Cite)"?**

Exactly, we have revisited this section to improve clarity and ensure the grouping logic is clearly conveyed. Lines 247-253.

**Lines 216-220: What exactly are you trying to say here?**

We have adjusted the text, now on lines 249-253 for clarity:

“This analysis evaluates whether species grouped by expected susceptibility-based groups exhibit consistent differences not only in period prevalence and cumulative mortality, but also in other epidemiological indicators throughout the course of the epizootic. The list of species by group is provided in Table 1.”

**Lines 227-229: condense: "Epi curves were generated from weekly tallies."**

We appreciate the suggestion and have condensed, now in lines 265-266.

**Lines 236-239: What is this association you are trying to make? How exactly did you compare density distribution of disease vs mortality? And why only compare density of disease between sites but nor mortality?**

Thank you for your observations. We have clarified the text to specify that the association being explored is whether mortality could help explain the observed incidence patterns. Additionally, we clarified that both incidence and mortality distributions were compared by site, not just incidence, as originally implied. Since this exploratory comparison was not part of the original study design, we did not conduct formal statistical tests, but aimed to highlight a potentially relevant pattern for future investigation. Thes explanations are now in lines 273 – 276:

“To explore whether mortality could help explain the observed incidence patterns, we visually compared the temporal alignment of daily incidence and mortality curves by overlaying their kernel density distributions. Additionally, to rule out the possibility that the pattern resulted from site aggregation, we compared incidence and mortality distributions by site.”

**Line 262: Earlier, you said epidemic curves were generated from weekly tallies. Now you say daily. Which is it?**

Thank you for your observation. As stated in lines 107–108 of the Introduction, we define epidemic curves specifically as incidence curves. And early in text we explained: “To highlight patterns in case emergence, epi-curves were constructed as weekly histograms.” (lines 265 – 266). In contrast, prevalence and cumulative mortality were calculated daily to track their progression throughout the epizootic. As stated in lines 297-298. Therefore, line 262 (now 299) remains as it was: “The prevalence was calculated daily as:”

**Lines 287-289: Delete. This does not add anything meaningful to previous sentence and overly complicates things and seems arbitrary (what is magical about 5%? why not 10%? 20%?).**

Thank you for your thoughtful observation. Our aim is to compare two approaches to disease detection: the time to the first observed case per species, and the time to reach a representative prevalence level. We have clarified the rationale in the manuscript, lines 323 – 327:

“In parallel, we estimated the time at which each species reached a prevalence of 5%, which we used as a population-level reference to compare detection dynamics. This value is informed by historical patterns in the Caribbean, where coral diseases typically remain below 5% under endemic conditions [85]. We hypothesize that traditional sampling methods tend to detect disease only after it exceeds such visibility thresholds, rather than capturing the earliest affected colonies.”

**Lines 291-298: Just how meaningful is this? Can you delete to simplify and get to the point?**

**Lines 299-326: Ditto.**

The two previous comments will be answered below.

Thank you for the suggestion. We understand the concern regarding length and clarity. That said, we agree that the presentation could be simplified, and we have revised the text to improve clarity. The new text is now between lines 328 and 352.

**Figure 2. Here it would be helpful to have actual N of colonies represented for each plot. I suspect for some plots (e.g. meandrina, dichocoenia,Porites), those are simply reflective of fact that those species are likely less numerous on reefs. Indeed, consider plotting curves only for species for which you have a minimal N (e.g. 20+) to make the data meaningful. Indeed, based on your data, I suspect susceptibility Table 1 is simply a function of how commonly those species are encountered and this could be a confounding factor (more disease simply because it is easier to detect and may not have anything to do with species susceptibility). Any way to correct for that? Indeed, a plot between relative numbers of each species at a site and the slope of population at risk (blue line) in your plot figure 2 might be informative. The closer the slope is to 0 or 1 suggests rare species which would be only those corals in intermediate slope values should really be considered when assessing epidemic curves.**

Thank you for the suggestion. We agree that sample size affects curve interpretability. To avoid overcrowding the figure, we have not added sample sizes (N) directly to each plot. However, these values are reported in Table 1 and will now be explicitly referenced in the figure legend to ensure clarity and transparency. We also discussed the potential influence of species abundance on curve shape and susceptibility metrics in the revised manuscript between lines 640-648:

“This study presents several methodological considerations: Variation in sample sizes across species, may influence the shape and duration of epidemic curves, particularly in underrepresented taxa. However, near-complete censuses within defined survey areas, recording all visible coral colonies regardless of species or condition, minimized sampling bias and reduced the likelihood of abundance-driven artefacts. Notably, *M. jacksoni* (N = 2) and *D. stokesii* (N = 6) showed extreme susceptibility (100% prevalence, mortality, and risk), consistent with reports from other regions [30, 33], while other low-abundance species such as *P. porites* (N = 18) and *I. sinuosa* (N = 8) exhibited much lower susceptibility (<13%). These patterns suggest that species-level vulnerability reflects intrinsic biological differences rather than sample size or visibility.”

**Figure 2 caption: "This is the point of time when the number of newly diseased individuals was maximum and then began to decline." Delete...redundant. Flashing line...do you mean solid black line?**

Thank you for the suggestion. We have removed that sentence. Regarding the line, we have changed it to "Dashed black lines".

**Figure 3. Consider deleting. This really does not add anything meaningful to your story and is redundant to Figure 2.**

We appreciate the suggestions; however, we retain Figure 3 as it clearly represents the approximate simultaneity in the progression of the epizootic across species groups. Which is one of the important findings of this research.

**Lines 404-407:"Furthermore, the species A. agaricites, A. tenuifolia, I. sinuous, S. intersepta, and P. porites exhibited low survival probabilities for their diseased populations (below 11 %), despite their at-risk populations showing moderate to high survival probabilities (S4 Fig)**

We appreciate the reviewer’s comment. We have modified the text to improve clarity.

**Figure 5 and 6: Delete. Does not really add anything meaningful to the story.**

We appreciate the recommendation. However, we believe both figures are important complements to the study. This figure illustrates the differences in risk and survival among species groups of susceptibility, which contrasts with the similarities observed in the epizootic progression across the same groups (Fig. 3). In turn, Figure 6 enables the exploration of species-specific susceptibility by integrating multiple epidemiological indicators. This integration reveals that some species exhibit higher-than-expected susceptibility; however, the overall trend remains consistent with susceptibility patterns reported in previous studies.

**Keep figure S2 and S3 and delete the rest. Those do not add anything meaningful to story.**

We appreciate the recommendation. However, we have decided to retain the figures, as they provide visual support for key statements in the manuscript. Figure S1 facilitates the understanding of the method described. Figure S4 highlights the variability in survival among species, a pattern referenced in the manuscript and reinforced by visual evidence. In turn, Figure S5 presents support for the hypothesis introduced at the outset, which constitutes one of the central pillars of this work.

**Lines 418-421: Delete. Not sure what this adds. What pairwise comparisons were different?**

Thank you for your comment. In the revised text, we clarified that post hoc pairwise comparisons revealed significant differences between species-specific groups. We retained these lines (now 436 – 439) because they highlight an ecologically relevant pattern: two species groups with different expected susceptibility showed similar dynamics during the early phase of the outbreak, despite statistical differences across the overall distribution of both curves. This reinforces the value of visual inspection (e.g., Figure 5) as a complement to statistical results.

**Lines 422-429: Condense to: "there were no statistically significant differences in survival probabilities among the diseased populations."**

Thanks for the recommendation, we have condensed the information in the paragraph. Now between lines 440 and 444.

**Lines 432-438: What is your point here? This paragraph makes no sense.**

Thank you for your observation. We revised the paragraph to clarify that daily prevalence curves may overestimate disease magnitude toward the end of the outbreak due to the declining number of at-risk colonies. What is remarkable in Figure 2. This effect is amplified by censoring events, which, when the at-risk population is already small, can disproportionately inflate prevalence estimates. To avoid this bias, we relied on period prevalence as a more robust and interpretable measure of severity during the final phase. This is now more clearly stated in the revised text between lines 447 and 452:

“The impact of the SCTLD epizootic on coral populations was assessed by period prevalence, cumulative mortality and peak incidence rate (height of peak; Table 1). Although daily prevalence curves increased toward the end of the outbreak in all species, this rise is partly driven by the declining number of at-risk colonies (Fig. 2), including censored individuals, which inflates daily prevalence values. Therefore, period prevalence was used as a more reliable measure of epizootic magnitude during the final phase.”

**Lines 439-444: Seems from FIgure 2 that prevalence peaked out at 1 for all species suggesting they are all equally susceptible no?**

We appreciate this observation. We would like to clarify that the prevalence axis is displayed on the secondary (right-hand) axis of each panel, as indicated in the figure legend, and is expressed as a percentage (0–100%). As shown, no species reached 100% prevalence at any point. We suspect that the confusion may stem from the cumulative mortality curve, which did reach 100% in some species. To avoid this potential misunderstanding, we have adjusted the line patterns of the curves in the revised figure.

**Lines 446-492: This is all very convoluted, and without an appreciation of underlying N for each species surveyed, it is difficult to sort out here whether these data are indicative of species susceptibility or simply an artefact that there is less disease evidence in species that are less visible or common.**

We thank the reviewer for this observation. As previously stated, the number of individuals (N) per species is provided in Table 1. Additionally, we have revised the entire section to improve clarity and flow. Now between lines 460 and 502. We also expanded the discussion to consider how differences in species abundance may influence the observed patterns.

**Line 457/Fig 6: Why are you splitting them on PC1? Why not PC2? Also, not understanding the rationale for the cutoffs for the 4 groups on PC1? Seems I could make my on grouping 1-4 based on PC quadrats. For example for outbreak length (PSTR, SSID, PCLI, MCAV), P survival (PAST, AAGA< OFAV, ATEN, OANN), etc. This all seems arbitrary to me.**

We thank the reviewer for this important observation. In our interpretation of the PCA, the vertical axis (PC2) is primarily influenced by outbreak duration, which, as discussed in the manuscript, may be affected by species abundance. We chose to include this variable in the PCA precisely because it helps visualize how outbreak duration may vary with species abundance, even if it does not directly reflect susceptibility. However, we based our classification on the horizontal axis (PC1), which captures the greatest spread of susceptibility-related parameters and explains 60.9% of the total variance. Therefore, we interpreted the gradient of susceptibility from right to left along PC1.

We did not use quadrant-based classification because relying on PC2 would imply lower susceptibility for species with longer outbreaks, which may simply reflect higher abundance. For instance, *P. strigosa* exhibited high risk and low survival, yet a quadrant-based approach would group it with species of moderate susceptibility such as *M. cavernosa*, whose proximity to the PCA center reflects a different epidemiological profile.

We will incorporate this rationale into the manuscript to clarify the basis of our classification in the lines 467 – 476:

“To achieve a more comprehensive understanding of species susceptibility, we integrated the assessed epidemiological parameters (Table 1) using a principal component analysis (PCA, Fig 6). This multivariate approach enabled us to position species along a susceptibility gradient, divided into four vertical segments (Levels I–IV) from right (highest susceptibility) to left (lowest). We based this classification on the horizontal axis (PC1), which explains 60.9% of the total variance and captures the widest distribution of susceptibility-related indicators (e.g., risk, survival, mortality, prevalence). In contrast, the vertical axis (PC2) is primarily influenced by outbreak duration, a variable that may be confounded by species abundance. Therefore, we avoided quadrant-based classification, which could misrepresent susceptibility in species with longer outbreaks but higher population sizes.”

**Lines 510-512: It is already known that there is difference in species susceptibility to SCTLD.**

We thank the reviewer for this comment. While it is true that species-level differences in susceptibility to SCTLD have been previously reported, to our knowledge no study has integrated epidemic risk and survival parameters to assess species-specific susceptibility in a multidimensional framework. The sentence in question aims to highlight that such susceptibility is not readily apparent in the temporal progression of the epizootic, but rather emerges when considering its magnitude, severity, and epidemiological risk.

**Reviewer #2:**

**This paper investigates the susceptibility of 16 species of stony corals based on epizootic and Kaplan-Meier curves, giving a picture of the incidence and spread over time that is much more accurate and comprehensive than punctual prevalence.**

**One concern I have is regarding how many colonies tissue loss was calculated based on a single observation and/or on extrapolating from algal colonization. Did this occur pretty evenly between species/ susceptibility classes, or did it disproportionally affect one or more and might have impacted those comparisons?**

We thank the reviewer for this thoughtful observation. A total of 218 colonies affected by SCTLD were analyzed using approaches III and IV, encompassing 14 of the 16 species. Among colonies with only a single record of tissue loss, 57% belonged to high susceptibility species, 44.5% to intermediate, and 62% to low susceptibility, proportions that are approximately balanced across susceptibility groups. However, a more influential factor was colony size: 61% of single-record cases corresponded to small colonies. We consider that this approach adds value by recovering information that would otherwise be excluded and by enabling the inclusion of these cases in broader epidemiological analyses. Nonetheless, we have revised the manuscript to clarify the assumptions and limitations associated with this inference. Lines 209 – 213:

“For colonies with only one recorded observation of tissue loss, we acknowledge that direct estimation of lesion progression is not possible. To avoid excluding these cases, we implemented two approaches based on biologically informed assumptions and empirical reference data. These methods aim to reconstruct plausible trajectories while accounting for uncertainty, as detailed in points III and IV.”

And in the discussion of the limitations. Lines 649 – 656:

“For colonies with only one recorded observation of tissue loss, inference-based approaches (III and IV, see methods) were applied transparently, incorporating ecological indicators and uncertainty. Among these single-record cases, the distribution across susceptibility groups was approximately balanced (57% high, 44.5% intermediate, 62% low; relative to diseased colonies in each group), suggesting no systematic bias toward any group. A more influential factor was colony size: 61% of these cases corresponded to small colonies. We consider that this approach adds value by recovering information that would otherwise be excluded and by enabling the inclusion of these cases in broader epidemiological analyses”

**I am interested to see the peaks in transmission following mortality, and speculation about the role of non-primary pathogens and dysbiosis. Could this explain the variety of histological lesions found in outbreaks after the early Florida investigation? Work et al. “Pathology of lesions in corals from the US Virgin Islands after emergence of stony coral tissue loss disease” describes a variety of lesions that largely do not match the ones found in the initial Landsburg paper.**

We thank the reviewer for this insightful comment. We have expanded the discussion to address the potential role of non-primary pathogens and dysbiosis in shaping the clinical presentation of SCTLD. Lines 583 – 586.

**Line 107: write 17^th^ century instead of using Roman numerals.**

Thank you for this recommendation. We have change it (Line 105).

**Lines 110-112: reads awkwardly, suggest: “They help to determine the progression, development and magnitude of an epidemic or epizootic, distinguish epizootic from enzootic phases, and can be compared with potentially correlated factors.”**

Thank you very much for the suggestion. We have modified the sentence for clarity (lines 108-110).

**Line 150: How many total surveys?**

**Write out “approximately” instead of using a tilde.**

Thank you for the suggestion. From June 2018 to July 2019, a total of 31 monitoring sessions were conducted between both sites. Stated in line 154.

**Line 150-153: “Both sites are 5 m deep, and the sites are separated by 1,100m” Or put the distance the sites are apart at the beginning of the paragraph “at two sites afflicted by SCTLD and 1,100 m apart in the Puerto Morales reef..”**

We appreciate the reviewer’s suggestion regarding the clarity of site descriptions. We have revised the paragraph to include the distance between sites earlier in the text, as recommended.

**Line 160: change to “During each subsequent survey”**

Thank you for the suggestion. We have changed the sentence (line 158-160).

**Line 161: How did you determine that SCTLD was the cause of tissue loss in any given colony? Was all observed tissue loss assumed to be due to SCTLD? Did you observe any loss to predation or other causes?**

Thank you for the questions. We observed lesions from predation or other diseases, such as black band, yellow band, or old mortality from unknown causes; these lesions were not considered SCTLD. We considered SCTLD when the tissue showed detachment, recent or non-old mortality, and no evidence of predation or other disease.

**Line 165: “focused”; maintain verb tense.**

Thank you for the suggestion. This section was revised to improve clarity. Line 173.

**Line 197: change “and” to “that” did not…**

Thank you for the suggestion. This section was revised to improve clarity. Line 228.

**Line 201/S5: How many species are included in the extrapolation of time since tissue loss using algal growth, and could this vary with different types/rugosity of skeleton between species?**

We thank the reviewer for this important observation. For these estimations we used a subsample of 157 colonies of seven species. We assumed that environmental factors would dominate over skeletal characteristics in driving secondary succession. However, we acknowledge that skeletal type and rugosity were not explicitly considered in the extrapolation. We have now included this point as a limitation of the method in the revised manuscript. Lines 659 -660:

“Finally, extrapolation of tissue loss timing via algal colonization did not account for skeletal morphology, though environmental conditions were assumed to dominate secondary succession.”

**Fig. 2, 3: The labels and numbers on these are blurry and very difficult to read in the PDF, although the downloaded figure is sharp. If this will be physically printed or published as a PDF a higher resolution image is needed.**

Thank you very much for your observation. We have improved image resolution.

**Line 608: change to “This is congruent with the finding that epizootics of this syndrome may not be dependent on colony density”.**

Thank you, that sentence has been modified for clarity. Line 595.

**Line 614: precisely? Or do you mean primarily.**

Thanks for the clarification. We meant that secondary waves begin to appear starting the cold season, but not exclusively. We've changed the wording for clarity. Line 601.

**Line 629: Don’t start the sentence with “and”; either “Additionally, histo…” or Just “Histopathologic analysis…”**

Thank you very much, we have added this correction. Line 617

**Line 652:”is not against” here is vague; do you mean “is not incompatible with” and “large” rather than “big”.**

Thank you very much, we have modified this sentence for greater clarity. Line 634.

**Reviewer #3:**

**I recommend that this manuscript needs significant revisions. This study addresses an important topic by applying epidemiological tools to coral disease; however, there are concerns regarding the limitations of the models and the results not supporting the claims in the discussion that need to be addressed before publication. Please see below for more comments and line by line suggestions.**

We thank the reviewer for their comments and have addressed the recommendations as detailed below.

**PONE-D-25-35026 Comments to Authors**

**General comments:**

**The conclusion that mortality appears to contribute to secondary transmission events should be more of a hypothesis as it is speculative. I suggest changing the terminology throughout the manuscript to soften the language as this is a hypothesis.**

We thank the reviewer for the recommendation. We have softened the statements to a more hypothetical tone in the abstract, lines 36 - 39:

“Temporal analyses revealed community-level patterns, including secondary outbreak waves following increases in mortality. This suggests a potential feedback mechanism, where mortality may contribute to secondary transmission events, a phenomenon not previously described in the study of coral epizootics.”

And in the lines 570–576:

“Previously, it has been raised that the dispersal of SCTLD could be favored by the sloughed tissue coming from dying colonies, as it is potentially loaded with microorganisms [15, 33, 53]. While our findings do not constitute direct field evidence, they provide model-based support for this hypothesis. Further investigation is needed to establish causality, but the observed patterns are consistent with a potential feedback loop in which coral tissue mortality contributes to increase transmission, thereby aggravating and prolonging the epizootic.”

**The figure descriptions are very long and also have some discussion points in them. I suggest removing the discussion points from the legends and including them in the manuscript if necessary (e.g., lines 104-105 are discussion points). Some of the figures are complicated, so potential reworking may be needed.**

Thank you very much for the recommendations. We have removed the discussion points from the figure captions and improved the wording for greater clarity.

**Point prevalence is the more commonly used term compared to punctual prevalence and should be changed throughout the manuscript.**

Thank you for the recommendation. We have replaced all mentions of “punctual” or “point prevalence” with “prevalence” alone, as reviewer #1 correctly noted that these terms are redundant in English. The specification “period prevalence” is retained only when referring to that particular metric.

**The limitations of the models need to be highlighted and discussed throughout the manuscript.**

We thank the reviewer for their thorough evaluation and constructive feedback. We discuss these limitations in the manuscript and have added a section on methodological considerations and limitations at the end of the discussion. Lines 639-660.

**General methods:**

**Though there was no difference in spatial distribution between sites, there needs to be evidence presented (I.e. supplementary statistics) that there are no incidence differences between sites. Since the authors are referring to temporal dynamics and not spatial distribution for these data, analyses need to be run to determine if the sites can be pooled.**

Thank you for your observation. We have added a more extensive justification for the decision to combine the site data in the manuscript. This can be found on lines 173 – 178:

“Given that our previous approximation did not detect spatial differences in SCTLD distribution between sites [26], cases from both sites were merged to increase the sample size. This broader approach is further supported by Sharp et al. [25], who found that SCTLD transmission may not be associated with the density of susceptible individuals, reinforcing the rationale for merging data from both sites to improve statistical power.”

**Why were only 422 corals used to estimate tissue loss per day when 990 had signs of SCTLD? It says further analyses were used for these 990, but more than half were not put into any of the models. Was a model applied to the other >500 corals? If so, which one was it applied to? That needs to be stated directly. If not used in this study, then those should be removed from the methods, table 1, discussion, etc.**

We thank the reviewer for pointing out this important clarification. The number 990 colonies refer to the total number of individuals belonging to the 16 susceptible species monitored during the study. However, only 442 of these colonies showed signs of SCTLD, and these are the ones included in the method’s section: “Individual Disease Status Reconstruction” (lines 180 – 238). This is clarified in lines 171 – 173:

“As a result of these surveys, a total of 1,007 colonies of 26 coral species were recorded. Among these, 990 colonies belonged to 16 species that exhibited SCTLD signs in at least one individual during the study period (n = 422). Further analyses were conducted on these 16 species.”

And 183-185:

“To achieve this, we reconstructed individual tissue loss trajectories using the percentage of dead tissue recorded for each affected colony (n = 422) at every monitoring event.”

**Models 3 and 4 – These need to be presented with more caution. Model 3 relies on observations of corals of the same species and size while Model 4 relies on a perceived timeline, and it needs to be addressed that these are less reliable methods to infer incidence.**

We appreciate the reviewer’s concern regarding Models 3 and 4. Both approaches were developed to address limited observations using biologically informed assumptions and empirical patterns. The manuscript has been revised to clarify these limitations and the rationale behind their use (lines 209 – 238). These approaches were also acknowledged in the final section of the discussion, where we outline methodological limitations and considerations for interpreting the results (lines 649 – 656).

**For the PCA – how were the variables scaled?**

We thank the reviewer for this observation. All variables included in the PCA were centered and scaled (subtracting the mean) and scaled to unit variance (dividing by the standard deviation) prior to analysis, using the scale. = TRUE argument in the prcomp() function of the *factoextra* R package. We have clarified this in this section of the methods. Lines 349 – 352.

**Species with a low sample size need to be addressed as a limitation of these models. For example, M. jacksoni is presented within figure 2, but only had a sample size of 2.**

While we acknowledge the limitations of low sample sizes, we believe that including all observed cases, regardless of abundance, provides a more complete picture of species-level susceptibility and disease progression. This approach avoids omission bias and supports future comparative and conservation efforts. Nonetheless, we have addressed the implications of low sample sizes in the discussion. Lines 640 – 648:

“This study presents several methodological considerations: Variation in sample sizes across species, may influence the shape and duration of epidemic curves, particularly in underrepresented taxa. However, near-complete censuses within defined survey areas, recording all visible coral colonies regardless of species or condition, minimized sampling bias and reduced the likelihood of abundance-driven artefacts. Notably, *M. jacksoni* (N = 2) and *D. stokesii* (N = 6) showed extreme susceptibility (100% prevalence, mortality, and risk), consistent with reports from other regions [30, 33], while other low-abundance species such as *P. porites* (N = 18) and *I. sinuosa* (N = 8) exhibited much lower susceptibility (<13%). These patterns suggest that species-level vulnerability reflects intrinsic biological differences rather than sample size or visibility”

**Figure 2 is overly complex, making it difficult to interpret. Consider simplifying or splitting into multiple panels.**

We appreciate your kind advice. We have kept the figure unchanged to avoid saturating the manuscript with additional visuals; however, we have improved its image quality.

**General discussion:**

**The structure of the discussion needs to be re-worked to have a better flow. Suggestion: summary of main findings, comparisons with previous work, interpretations of key patterns, limitations, implications for future use.**

We appreciate your recommendations and have restructured the discussion to improve flow and clarity, adding dedicated sections on limitations and implications.

**The language has to be softened within this section. For example, “point prevalence may not always be a reliable indicator of susceptibility, especially when measured at a single time point.”**

We appreciate the recommendation and have softened the statements.

**The PCA provides an exploratory framework for susceptibility, and it complements existing classifications, but tone down the language regarding the novel classification.**

Thank you very much for the recommendation, we will tone down the language regarding the new classification.

**In general, the language regarding the “secondary waves caused by sloughed off tissue” needs to be softened as it is a hypothesis and cannot be proven with modeling.**

We appreciate the recommendation; we have clarified that it is a hypothesis based on our observations.

**It is unusual to have phrased questions throughout – consider revising for easier reading.**

We thank the reviewer for this stylistic observation. We recognize that the use of phrased questions is uncommon in scientific writing and may affect the flow or clarity of the discussion. We have revised the manuscript to rephrase rhetorical questions as direct statements.

**Lines 636-650 – it is unclear why there is significant detail regarding opportunistic infections and the coral’s response. I suggest cutting down on these sections as they have been described in other studies and do not necessarily discuss the results of this study. Or potentially adjust the flow of the discussion so that the interpretation of susceptibility differing is clearer.**

We appreciate the reviewer’s observation regarding the level of detail on opportunistic infections and coral immune responses. In response, we have substantially condensed this section to better align with the scope of our study. The revised paragraph (lines 624 – 632) now focuses on the mechanistic plausibility of opportunistic infections contributing to differential susceptibility, without reiterating background information extensively covered elsewhere. This adjustment also clarifies the link between microbial disruption, immune dysregulation, and symbiont resilience, which supports our interpretation of host-specific recovery potential.

**I highly suggest adding a limitations section to the discussion where the drawbacks of the models are discussed. For example, limitations include variation in sample size, inferred incidence for Models 3 and 4, pooling sites, non-independence of colonies, etc.**

Thank you very much for the recommendation, we have added this section to the discussion between lines 639 and 660.

**Consider adding a final paragraph discussing the implications of the modeling and how they can be used in the future. Suggestions include for future outbreaks, provide targeted interventions, aid mangagement decisions, etc.**

Thank you very much for this recommendation, which we have taken into account in the restructuring of this section of the manuscript. Lines 661-675:

“This study highlights the value of epidemic curves as a central tool for characterizing coral disease dynamics. Their ability to capture the temporal structure of outbreaks makes them especially useful for detecting shifts in disease behavior and identifying outbreaks that may escalate into epizootics. Given their ease of implementation and interpretability, epi-curves can be integrated into routine monitoring programs to provide real-time insights and support timely decisions on surveillance, containment, and restoration. When combined with risk-based indicators, they offer a robust framework for adaptive management, helping practitioners anticipate critical transitions and prioritize interventions. Although applied here during an active outbreak, this approach can be extended to long-term monitoring, generating high-resolution temporal data that reveals subtle changes in disease dynamics and enable links to environmental or anthropogenic drivers. Moreover, the use of epidemic curves and integrated indicators is scalable to other coral diseases and marine taxa affected by epizootic events. As SCTLD continues to expand across the Caribbean, these tools could enhance regional coordination, optimize resource allocation, and strengthen conservation responses.”

**Finally, consider adding Aeby et al 2025 “Progressive chronic tissue loss disease in Siderastrea siderea on Florida’s coral reef” as a potential explanation for the Sids species susceptibility differences.**

Thank you for the recommendation, we have included Aeby et al., 2025.

**Line by line suggestions:**

**Line 38 – mortality may contribute to secondary transmission**

Thank you for the recommendation, we have modified the sentence. Line 38.

**Line 41 – say what you are comparing the higher level of susceptibility to – i.e., previous studies**

Thank you very much, we have clarified that greater susceptibility will be observed in these species compared to previous studies. Line 43.

**Line 51 – sensible doesn’t fit here. Sensitive? Susceptible?**

Thank you very much for the recommendation, we have opted for “vulnerable” which emphasizes susceptibility to disturbances. Line 51.

**Line 58 – stony coral tissue loss disease is lowercase**

We thank the reviewer for the observation. We have been corrected to lowercase as suggested.

**Lines 73 – 76 – the wording is confusing to read. Revise the “will also have likely compromised.” Potentially break into two sentences as well for clarity**

We appreciate the reviewer’s suggestion and have revised the sentence for clarity by simplifying the structure and separating key ideas. Lines 73 -76.

**Line 79 – Change “punctual prevalence” to “point prevalence” throughout the manuscript as it is more the more commonly used term.**

We appreciate the observation and have replaced all mentions of “punctual prevalence” with “prevalence” as previously noted.

**Line 84 – change “lesions apparition” to “lesion appearance” if that maintains the meaning of the sentence**

Thank you very much for the recommendation. We changed it to “lesion appearance”. Line 83.

**Line 88 - “giving an incomplete picture” is awkward phrasing for the sentence. Consider revising**

We thank the reviewer for the suggestion. The sentence has been revised to improve clarity. Line 87.

**Line 100 figure 1 description – there are no white arrows within the figure, so assuming you are referring to black arrows. Remove discussion/result points from the figure descriptions.**

Thank you very much for this observation. As the reviewer noted, the arrows are black, not white. We have adjusted the legend and removed the discussion points.

**Line 111 – Revise sentence to avoid starting with “which.” Potentially combine with previous sentence.**

Thank you very much for the comment. We have restructured both sentences to avoid using "which" at the beginning.

**Line 137 – Consider “epi-curves, Kaplan-Meier risk curves, and survival curves” if it maintains meaning.**

Thank you for the recommendation. We change to: “epi-curves and Kaplan-Meier risk and survival analyses” (line 135-136).

**Line 182 – Repeated measures within the same individuals may violate independence assumptions of some models and should be stated that it should be interpreted with caution.**

We appreciate the reviewer’s observation. To account for repeated measures within individuals, we employed Generalized Additive Mixed Models (GAMMs) with autocorrelation adjustments, which do not assume independence between observations. Moreover, repeated measures were used exclusively to reconstruct individual tissue mortality trajectories, and not for population-level inference, thereby minimizing concerns regarding independence violations.

**Line 219 – change to epizootic**

Thank you very much, we now use "epizootic".

**Lines 233-236 – the language here needs to be softened as correlation does not mean causation, and this is a hypothesis**

We thank the reviewer for the feedback. We have softened the sentence and integrated the hypothesis discourse into the stated relationship. Lines 270 – 273:

“After observing secondary waves in the combined epi-curve, we hypothesized that these might be linked to the dispersal of the causative agent from sloughed tissue of dying colonies, which are potentially laden with microorganisms that could contribute to the feedback of the epizootic [30, 33, 53].”

**Line 270-273 – revise sentence for readability**

Thank you for the recommendation, we have improved the clarity of the sentence. Lines 307-309.

**Line 289 – change “umbral” to “threshold”**

Thank you very much, we have rewording the sentence (lines 325-326).

**Line 292 – change to “on the other hand”**

Thank you, the section has been reconfigured for greater clarity. Lines 329-335.

**Line 339 figure 2 description – “flashing is unclear” changed to dashed or change the figure so that the dotted and dashed black lines are easier to differentiate**

Thank you very much for the recommendation, we now use “dashed” and although we maintain the line pattern, we have increased the difference between both line patterns.

**Lines 357 – 371 – the sample size of the corals needs to be clearly addressed when comparing between species.**

We thank the reviewer for the recommendation. We have now incorporated explicit mentions of sample sizes per species within this section. Lines 375 – 392.

**Line 381-382 – revise for clarity**

Thank you very much, we have improved the clarity of the sentence. Lines 402 – 403.

**Lines 392-393 – revise to “this patten is consistent with the hypothesis” as it does not prove it. It is not possible to confirm that sloughing off tissue is causing a secondary wave with these models alone.**

Thank you very much for the recommendation. Since this is a point of discussion, we have decided to remove this sentence and leave only the figure description.

**Line 409 figure 5 description – revise for clarity about B and C**

Thank you very much, we have improved the clarity regarding B and C. Lines 427 – 433.

**Line 429 – change to log rank**

We appreciate the recommendation. However, the Kruskal–Wallis (K-W) test was used specifically to compare survival values at the end of follow-up among species grouped by susceptibility. In contrast, the log-rank test was applied to assess differences across the entire survival distribution. We have clarify this in the text, lines 442-445:

“However, differences among diseased populations were smaller (χ2 = 62.5 vs. 296; Fig 5C), and by the end of the follow-up, when all unrecovered colonies had died, no significant differences remained (K–W test, p = 0.22).”

**Line 471 – revise for clarity**

Thank you very much, we have reviewed the entire section to improve clarity. Lines 486 – 502.

**Figure 6 and description – the four “levels” of susceptibility are not based on formal clustering, which makes this classification subjective. Reframe to be exploratory instead of discrete groups. Clarify how the variables were standardized before the PCA. The legend is dense and hard to follow, so the text needs to be simplified. The results are interesting, but the interpretation should be more cautious and should be presented as exploratory rather than definitive evidence of a new susceptibility classification.**

We appreciate the reviewer’s observations. The four susceptibility levels were not derived from formal clustering algorithms, but rather represent exploratory groupings based on species’ positions in PCA space and their observed epidemiological trajectories. We have revised the text to clarify that these levels are intended as interpretative tools to highlight emerging patterns, not as definitive categories. Lines 137 – 139:

“Furthermore, by integrating the assessed epidemiological parameters into a multi-dimensional space, we outline a four-level framework that contributes to the characterization of species susceptibility to SCTLD.”

We now explicitly state that all variables included in the PCA were standardized (centered and scaled to unit variance) prior to analysis.

The legend of Figure 6 has been simplified to improve readability, and the interpretation of results has been reframed to emphasize their exploratory nature rather than suggesting a formal classification scheme. Lines 481 – 485.

**Lines 510-512 – revise for clarity**

Thank you, we have reviewed the sentence to make it clearer. Lines 518 – 520.

**Line 512 – species' if it maintains clarity**

Thank you for the suggestion. In this case, we retained “species groups” without the possessive, as it refers to groups defined by species characteristics rather than groups belonging to the species.

**Line 527 – point prevalence may not be a reliable measure**

Thank you, we have softened the sentence using your recommendation. Line 537.

**Line 557 – outbreak duration may not be a reliable measure**

Thank you, we have softened the sentence using your recommendation. Lines 534 – 535.

**Lines 574-575 – revise for clarity**

Thank you, we have modified the sentence to improve clarity. Lines 566-567.

**Lines 581 – 582 – soften this language as this is not direct evidence from the field and these observations need experimental manipulations instead of modeling to prove causation and not just correlation**

Thank you for your observation. We have revised the text to clarify that our findings do not constitute direct field evidence but rather provide model-based support for the hypothesis. We now emphasize the need for experimental validation to establish causality. Lines 570 – 576:

“Previously, it has been raised that the dispersal of SCTLD could be favored by the sloughed tissue coming from dying colonies, as it is potentially loaded with microorganisms [15, 33, 53]. While our findings do not constitute direct field evidence, they provide model-based support for this hypothesis. Further investigation is needed to establish causality, but the observed patterns are consistent with a potential feedback loop in which coral tissue mortality contributes to increase transmission, thereby aggravating and prolonging the epizootic.”

**Line 608 – revise so the sentence does not start with which**

Thank you, we have modified the sentence. Line 593.

**Lines 610-619 – add Palacaio-Castro “elevated temperature decreases stony coral tissue loss disease transmission, with little effect of nutrients” to discussion**

Thank you for the suggestion. However, we believe that the context of the experiment in the cited work does not correspond to our analysis of transmission during epizootics within a broader ecosystem-level framework of complexity, although it does offer an interesting insight into a counterintuitive effect of temperature on SCTLD transmission at mesoscale.

**Line 652 - “is not against” is unclear to read**

Thank you, we have modified the sentence for greater clarity. Line 634.

**Line 671 – misused may not be the appropriate term here**

Thank you, we have softened the language in this sentence. Line 679.

**Line 672-674 – soften the language here as the models could aid in elucidating the etiology of SCTLD**

Thank you, we have softened the language in this sentence. Lines 780 – 781.
